# Clinical Characteristics of Patients with Newly Diagnosed Diabetic Macular Edema in Turkey: A Real-Life Registry Study—TURK-DEM

**DOI:** 10.1155/2017/3596817

**Published:** 2017-02-19

**Authors:** Bora Eldem, Sengul Ozdek, Ali Osman Saatci, Emin Ozmert, Esat Ulay, Gulsah Nomak

**Affiliations:** ^1^Department of Ophthalmology, Hacettepe University Faculty of Medicine, Ankara, Turkey; ^2^Department of Ophthalmology, Gazi University Faculty of Medicine, Ankara, Turkey; ^3^Department of Ophthalmology, Dokuz Eylul University Faculty of Medicine, Izmir, Turkey; ^4^Department of Ophthalmology, Ankara University Faculty of Medicine, Ankara, Turkey; ^5^Bayer Türk, Istanbul, Turkey

## Abstract

*Purpose.* To evaluate the clinical and diagnostic characteristics of patients with newly diagnosed diabetic macular edema (DME) in Turkey in a real-life setting. *Methods.* A total of 945 consecutive patients (mean (SD) age: 61.3 (9.9) years, 55.2% male) with newly diagnosed DME were included. Data on patient demographics, comorbidities, ocular history, ophthalmic examination findings including type of DME, central macular thickness (CMT) via time domain (TD) and spectral domain (SD) optical coherence tomography (OCT), and planned treatments were recorded. *Results.* OCT (98.8%) and fundoscopy (92.9%) were the two most common diagnostic methods. Diffuse and focal DMEs were detected in 39.2% and 36.9% of cases, respectively. Laser photocoagulation (32.1%) and antivascular endothelial growth factors (anti-VEGF; 31.8%) were the most commonly planned treatments. The median CMT in the right eye was significantly greater in untreated than in treated patients [376.5 *μ*m (range: 160–840) versus 342 *μ*m (range: 146–999) (*p* = 0.002)] and in the left eye [370 *μ*m (range: 201–780) versus 329 *μ*m (range: 148–999) (*p* < 0.001)]. *Conclusions.* This study is the first large-scale real-life registry of DME patients in Turkey. SD-OCT and fundoscopy were the most common diagnostic methods. Laser photocoagulation and anti-VEGF therapy were the most common treatments.

## 1. Introduction

Diabetic macular edema (DME) is a major complication of diabetes mellitus that affects central vision. The symptoms of DME range from slight visual blurring to complete blindness if left untreated [1, 2]. Recent estimates are that, by the year 2030, 350 million people worldwide will have diabetes [3] and 100 million of them will have DME [4].

The worldwide prevalence of DME was reported as 6.81% among patients with diabetes [5]. Other research reported that the 10-year incidence of DME was 14% in individuals with type 2 diabetes, and progression to DME occurred in 29% of individuals with type 1 diabetes over 25 years if left untreated [6, 7]. Although DME resolves spontaneously in about 33% to 35% of patients, it tends to be chronic in most patients [3, 8, 9]. DME accounts for the loss of 3 Snellen lines of vision in 24% of eyes within 3 years [8] and in 12% of all new cases of blindness annually [10]. The high prevalence and potential severity of DME point to the need for prompt and effective treatment [11, 12].

Limited data are available on characteristics of DME patients in Turkey, and most data are from clinical trials. Only one multicenter registry examined the frequency of diabetic retinopathy and risk factors in Turkey. This previous study reported that the overall prevalence of DME was 3.4% in patients with newly diagnosed diabetes (*n* = 298), 9.7% in those with type 1 diabetes and 2.4% in those with type 2 diabetes [13].

Real-life studies have high generalizability because, in contrast to randomized controlled trials, they provide data on real-life situations rather than on a specific set of patients who were selected under strict and controlled conditions [14]. There is a need for a registry study to investigate the baseline characteristics of patients with DME in Turkey. The TURK-DEM study is the first large-scale observational registry of DME patients in Turkey. The purpose is to evaluate the real-life demographic, clinical, and diagnostic characteristics of patients with newly diagnosed DME in Turkey.

## 2. Material and Methods

### 2.1. Study Population

A total of 945 consecutive patients (mean age ± SD: 61.3 ± 9.9 years, 55.2% male) with newly diagnosed DME were included in this national, multicenter, cross-sectional, noninterventional, observational, and single-visit study conducted at 36 retina centers across Turkey between March 2013 and July 2014. Study centers were well-equipped reference centers and were selected so that the study patients had geographical and other characteristics that were representative of Turkey in general.

All patients were at least 18 years old, had newly diagnosed DME, and received no previous DME-specific treatment. Patients who participated in a previous clinical study and those who already received or were currently receiving treatment for DME were excluded.

Each subject provided written informed consent for participation after being provided with a detailed explanation of the study objectives. The protocol of this study was approved by the institutional ethics committee and was conducted in accordance with the ethical principles stated in the “Declaration of Helsinki” and local regulations.

### 2.2. Data Collection

All patients were evaluated on a single visit, and data on demographics and medical history were recorded. This included history of diabetes (type, treatment, presence of systemic complications such as diabetic foot, time from onset to DME diagnosis, and follow-up care), hypertension and receipt of antihypertensive treatment, vital signs, body mass index (kg/m^2^), blood biochemistry, ocular history (reason for visiting an ophthalmologist, concomitant ocular diseases, and previous eye operations), ophthalmic examination findings including Early Diabetic Retinopathy Study (ETDRS), best-corrected visual acuity (BCVA), Snellen test score, type of DME (focal, diffuse, center-involving, clinically significant, and others), central macular thickness [CMT at the time of diagnosis via time domain (TD) and spectral domain (SD) optical coherence tomography (OCT)], and planned pharmacological treatments (corticosteroids, antivascular endothelial growth factor [anti-VEGF] agents) and nonpharmacological treatments (laser photocoagulation, vitrectomy).

### 2.3. Statistical Analysis

Sample size calculation was performed using NCSS PASS 11 ver. 11.0.7 (Utah, USA). The results indicated that at least 900 patients from 36 centers should be included to achieve a confidence level of more than 95% and a statistical power of 80%, based on the patient enrollment capacity of the study centers (40 patients/year in high-capacity centers, 13 patients/year in low-capacity centers).

Statistical analysis was performed using SPSS (IBM Corp. 2012, IBM SPSS Statistics for Windows, version 21.0., Armonk, NY). When the data had nonnormal distributions, the Mann-Whitney *U* test was used to compare paired independent groups and the Kruskal-Wallis test was used for multiple comparisons of independent groups. In subgroup comparisons, when a nonnormal distribution was present, the Mann-Whitney *U* test, chi-square test, and Fisher's exact test were performed with the Bonferroni correction. For categorical variables, when the conditions for a chi-square test were present, the chi-square test was used to analyze paired and multiple groups. When the conditions for a chi-square condition were not present, Monte Carlo simulation was used for multiple group comparisons. All data are expressed as means and standard deviations (SDs), medians and minimum–maximum values, or percentages where appropriate. A *p* value less than 0.05 was considered statistically significant.

## 3. Results

### 3.1. Baseline Characteristics

We enrolled 945 consecutive patients [mean (SD) age: 61.3 (9.9) years, 55.2% male], 96.4% with type 2 diabetes and 3.6% with type 1 diabetes (Table 1). The time from onset of diabetes to diagnosis of DME was 10–14 years in 29.5% of patients and was less than 5 years in 12.0% of patients. Most patients were receiving treatment for diabetes (65.5%), and most were followed by internal medicine clinics (47%). Data on blood biochemistry were available for 263 patients (27.8%). These data indicate that the mean (SD) fasting blood glucose level was 187.8 (81.9) mg/dL and HbA1c level was 8.8 (2.3) %.

### 3.2. Ocular History

Most patients visited an ophthalmologist due to vision problems (52.2%) rather than due to referral (Table 2). The most common concomitant eye disease was cataract (29.9%). Overall, 162 patients (17.1%) had previous ocular operations and there were 210 total eye operations (due to multiple procedures in some patients). Cataract surgery was the most common eye operation (*n* = 210, 92.4%).

### 3.3. Ophthalmic Findings

During the study visit, fundoscopy was performed in 878 patients (92.9%) and fundus fluorescein angiography in 690 patients (73.0%) (Table 3). OCT was performed in 935 patients (98.8%); SD-OCT was performed in 838 patients (89.6%) and TD-OCT in 97 patients (10.4%). Overall, 11 patients were only assessed using a slit-lamp with a +78 D lens for diagnosis of DME.

Intraocular pressure was measured in 655 patients (69.3%); this was determined by a pneumatic method in 459 patients (70.1%) and an applanation method in 196 patients (29.9%). The mean (SD) intraocular pressure was 15.5 (3.3) mmHg in the right eye and 15.7 (3.8) mmHg in the left eye (Table 3).

The BCVA was measured using an ETDRS chart in 36 right eyes and in 35 left eyes and using a Snellen chart in 771 right eyes and in 769 left eyes. The mean (SD) ETDRS-BCVA was 63.3 (58.5) logMAR for the right eye and 68.4 (58.1) logMAR for the left eye in 36 patients. The mean (SD) Snellen score was 0.5 (0.3) for both eyes in 771 and in 769 patients (Table 3).

Overall, 1888 eyes were examined. Diffuse DME was diagnosed in 39.2% of eyes and focal DME in 36.9% of eyes. At the time of diagnosis, the mean (SD) CMT was 381.1 ± 140.4 *μ*m for both eyes (*n* = 1183) and a CMT greater than 300 *μ*m was present in 66.6% of eyes. At diagnosis, the mean (SD) CMT of the right and left eyes was 384.6 (144.0) *μ*m and 377.7 (136.6) *μ*m, respectively. The mean (SD) CMT of the right and left eyes was 341.6 (129) *μ*m and 342.0 (124.7) *μ*m based on TD-OCT and 389.3 (145.2) *μ*m and 381.5 (137.6) *μ*m based on SD-OCT (Table 3).

### 3.4. Treatment Preferences

Laser photocoagulation therapy (32.1%) and anti-VEGF therapy (31.8%) were the most commonly preferred planned treatments, followed by anti-VEGF + laser photocoagulation therapy (30.8%) (Table 4).

### 3.5. Clinical Characteristics of Patients with or without Antidiabetic Treatment

Patients who received antidiabetic treatment were significantly more likely to have type 1 diabetes (*p* = 0.005), to have a longer duration of diabetes (*p* < 0.001), to be active smokers (*p* < 0.001), and to have hypertension (*p* < 0.001) and dyslipidemia (*p* < 0.001) (Table 5).

Patients who were not treated for diabetes were significantly more likely to have diffuse DME in the right eye (*p* < 0.001) and a CMT greater than 300 *μ*m in both eyes (*p* < 0.001 for each). In addition, the median CMT was significantly greater in untreated than that in treated patients with diabetes in the right eye (376.5 versus 342 *μ*m, *p* = 0.002) and in the left eye (370 versus 329 *μ*m, *p* < 0.001) (Table 5).

## 4. Discussion

The present TURK-DEM study is the first large-scale real-life observational clinical study of patients with newly diagnosed DME in Turkey. In line with the previous observation that DME has a higher prevalence in patients with type 2 diabetes than type 1 diabetes [15], most of our study population had type 2 diabetes. Only 65.5% of our patients were receiving antidiabetic treatment, and the average HbA1c level was 8.8%. This seems notable given the previously reported association of elevated levels of HbA1c with DME prevalence and deterioration, particularly for patients whose HbA1c level is above 7% and who have had diabetes for a long time [1, 16, 17].

The time from diagnosis of diabetes to occurrence of DME was 10–19 years in half of recruited patients and less than 5 years in only 12.0% of the patients [18]. This supports the previously reported increase in the prevalence of DME with longer duration of diabetes mellitus [13, 18].

In line with the previously reported high prevalence of hypertension in patients with DME [19], 55.1% of our patients had hypertension and 77.5% of these hypertensive patients were being treated with antihypertensive agents. Given the increased risk for development and progression of diabetic retinopathy among diabetics with poorly controlled hypertension [20] and the greater risk for development of DME in the presence of hypertension [19], the findings emphasize the importance of controlling hypertension for the prevention and management of DME.

Ophthalmologists and physicians from other disciplines who care for patients with diabetes have an increased awareness of recent advances in DME management, and this may have helped to maximize the impact of these advances [21]. In this cohort, vision problems rather than referral were the most frequent reason for initial admission. Thus, it seems necessary to establish an efficient referral system to enable DME to be diagnosed at an earlier stage.

OCT was the most common method used for diagnosis of DME in our cohort (98.8%), followed by fundoscopy (92.9%). SD-OCT was used in most cases, and TD-OCT was only used in 10% of our patients. This seems notable given that pattern of edema classification on SD-OCT can have a significant impact on treatment decisions and subsequent visual outcome [22–25]. Also, the difference between CMT measurements obtained via TD-OCT and SD-OCT in our study is consistent with previous reports that CMT measured by SD-OCT is 45 *μ*m to 58.5 *μ*m greater than that measured by TD-OCT [26–28]. Hence, even though the results of these two methods are strongly correlated, our findings support the view that careful consideration should be given to data on CMT measurements that are determined by different OCT methods [26–28]. Regarding CMT values, previous research reported that eyes with greater baseline CMT levels (>400 *μ*m versus <300–400 *μ*m) had greater improvement of visual acuity in patients treated with an intravitreal anti-VEGF agent [29, 30].

A previous case series indicated that diffuse DME was refractory to macular laser photocoagulation therapy [31]. Thus, the presence of diffuse DME in almost half of the recruited patients is notable given that therapy for diffuse DME remains a major challenge [32].

Moreover, we found that 66.6% of the patients had baseline CMTs greater than 300 *μ*m, patients with diffuse DME and with a higher baseline CMT (>300 *μ*m) were less likely to have received antidiabetic treatment, and there were significantly higher CMTs in untreated than treated patients with diabetes.

The two major nonsurgical treatments for diabetic retinopathy are retinal laser photocoagulation and pharmacologic approaches, including corticosteroids and VEGF inhibitors [33, 34]. Several biodegradable and nonbiodegradable delivery systems can also help to achieve sustained levels of corticosteroids in the vitreous cavity [35]. Anti-VEGF therapy with pegaptanib sodium, ranibizumab, bevacizumab, or aflibercept can reduce edema and central retinal thickening and improve vision gain or stability [29, 36–39]. Laser photocoagulation is still considered one of the mainstays of treatment for DME along with other alternatives, especially focal DME [17]. Moreover, use of laser therapy after sufficient thinning of the retina by anti-VEGF drugs can promote stabilization of retinal thickness and reduced treatment burden, thereby improving functional outcome and reducing the need for further anti-VEGF injections [17, 40]. Consistent with these data, laser therapy and anti-VEGF drugs—either alone or in combination—were used to treat most of the cases in our cohort. Thus, the diagnostic work-up and practice patterns among Turkish DME patients seem to be in accordance with recent trends in the management of DME, including the common use of OCT for diagnosis, and the change of standard treatment from laser photocoagulation therapy to intraocular delivery of anti-VEGF agents [21].

The major strength of this observational study is that we examined the records of 945 patients with DME from 36 centers throughout Turkey. This means that our findings are probably generalizable to the overall population of Turkey. The main limitation of our study is its observational design, because nonrandomized allocation might have led to bias and confounding. Nevertheless, given the paucity of reliable information on DME in Turkey, our findings provide important baseline data for a large representative sample of DME patients from Turkey and thus constitute a valuable contribution. More importantly, this study is the most comprehensive real-life investigation of newly diagnosed DME that employed detailed analysis of baseline characteristics and of the different tools and techniques used for diagnosis.

## 5. Conclusion

In conclusion, the present study is the first large-scale real-life registry of newly diagnosed DME patients in Turkey. Most of the patients in our cohort had type 2 diabetes, poor glycemic control, and concomitant systemic hypertension and were diagnosed with diabetes 10–19 years previously. A vision problem, rather than referral from another physician, was the most frequent reason for seeking care from an ophthalmologist. SD-OCT and fundoscopy were the most common diagnostic methods, and laser therapy and anti-VEGF therapy were the most common treatments. Two-thirds of patients had baseline CMT values greater than 300 *μ*m, and almost half of the patients had diffuse DME. A baseline CMT greater than 300 *μ*m and diffuse DME were more common in those not receiving treatment for diabetes. The baseline data provided by the present registry provides a foundation for future studies of DME screening and treatment. It also could be used as a basis for prediction of treatment success and the likelihood of reducing visual impairment due to diabetes based on different patient characteristics.

## Figures and Tables

**Table 1:** Baseline characteristics of patients from Turkey with newly diagnosed diabetic macular edema.

*Age, mean (SD; min–max)*	61.3 (9.9; 20–88)
*Sex, n (%)*	
Male	522 (55.2)
Female	423 (44.8)
*Body mass index (kg/m* ^*2*^ *), mean (SD)*	28.9 (5.4)
*Systolic blood pressure (mmHg), mean (SD)*	132.3 (15.6)
*Diastolic blood pressure (mmHg), mean (SD)*	83.5 (11.6)
*Hypertension*	
Present	521 (55.1)
Undertreatment^a^	404 (77.5)
On dialysis	13 (5.7)
*Diabetes*	
Type 1	34 (3.6)
Type 2	911 (96.4)
Undertreatment^b^	619 (65.5)
Diabetic foot disease	44 (4.7)
*Time from onset of diabetes to DME diagnosis, n (%)*	
Less than 5 years	113 (12.0)
5–9 years	200 (21.2)
10–14 years	279 (29.5)
15–19 years	193 (20.4)
20 years +	160 (16.9)
*Disciplines involved in diabetes follow-up care, n (%)*	
None (not followed up)	165 (17.5)
Endocrinology	267 (28.3)
Internal medicine	444 (47.0)
Family practice	65 (6.9)
Nephrology	4 (0.4)
*Blood biochemistry (n* = 263)	
Fasting blood glucose (mg/dL)	
*N*	206
Mean (SD)	187.8 (81.9)
HbA1c (%)		
*N*	165
Mean (SD)	8.8 (2.3)
Total cholesterol (mg/dL)	
*N*	99
Mean (SD)	203.7 (61.2)
LDL (mg/dL)	
*N*	114
Mean (SD)	124.5 (42.0)
HDL (mg/dL)	
*N*	98
Mean (SD)	47.7 (21.3)
Triglyceride (mg/dL)	
*N*	113
Median (min–max)	142 (43–880)
Urea (mg/dL)	
*N*	127
Median (min–max)	28.8 (4.4–178)
Creatinine (mg/dL)	
*N*	140
Median (min–max)	0.9 (0.4–7.0)
Microalbuminuria (mg/dL)	
*N*	36
Median (min–max)	25 (0–741)

Missing data for ^a^27 and ^b^130 patients.

**Table 2:** Ocular history of patients from Turkey with newly diagnosed diabetic macular edema.

*Reason for visiting ophthalmologist*	*n (%)*
Routine control	360 (38.1)
Vision problem	493 (52.2)
Consultation	92 (9.7)
*Referred from (for routine control or consultation)*	
No referral	672 (71.1)
Endocrinology	108 (11.4)
Internal medicine	107 (11.3)
Family medicine	5 (0.5)
Other	53 (5.6)
*Concomitant eye diseases, n (%)*	
Cataract	283 (29.9)
Glaucoma	36 (3.8)
Other	626 (66.2)
*Previous eye operations, n (%)*	162 (17.1)
*Total eye operations*	210
Cataract surgery	194 (92.4)
Vitrectomy	4 (1.9)
Fellow eye operations	12 (5.7)

**Table 3:** Ophthalmic examination findings of patients.

	Right eye	Left eye		
*ETDRS best-corrected visual acuity score*				
*N*	36	35		
Mean (SD)	63.3 (58.5)	68.4 (58.1)		
Median (min–max)	56.5 (0–310)	55 (0–280)		
*Snellen test score*				
*N*	771	769		
Mean (SD)	0.5 (0.3)	0.5 (0.3)		
Median (min–max)	0.5 (0.05–1)	0.5 (0.05–1)		
*n* (%)				
1.0 logMAR (20/200)	97 (12.6)	92 (12)		
0.9 logMAR (20/158)	43 (5.6)	44 (5.7)		
0.8 logMAR (20/126)	58 (7.5)	69 (9)		
0.7 logMAR (20/100)	69 (8.9)	61 (7.9)		
0.6 logMAR (20/79)	61 (7.9)	58 (7.5)		
0.5 logMAR (20/63)	76 (9.9)	73 (9.5)		
0.4 logMAR (20/50)	59 (7.7)	71 (9.2)		
0.3 logMAR (20/39)	67 (8.7)	75 (9.8)		
0.2 logMAR (20/31)	81 (10.5)	78 (10.1)		
0.1 logMAR (20/25)	75 (9.7)	61 (7.9)		
<0.1 logMAR (20/22)	85 (11)	87 (11.3)		
*Intraocular pressure (mmHg), mean (SD)*	15.5 (3.3)	15.7 (3.8)		

DME type, *n* (%)	Right eye	Left eye		Both eyes

Diffuse	403 (42.7)	337 (35.7)		740 (39.2)
Focal	325 (34.4)	371 (39.3)		696 (36.9)
Mix	93 (9.9)	89 (9.4)		182 (9.6)
Not defined	120 (12.7)	130 (13.8)		250 (13.2)
Invisible fundus	3 (0.3)	17 (1.8)		20 (1.1)
Total^a^	944 (100.0)	944 (100.0)		1888 (100.0)

Central macular thickness (*μ*m)^b^	Right eye	Left eye		Both eyes

At the time of diagnosis				
*N*	912	901		1813
Mean (SD)	384.6 (144.0)	377.7 (136.6)		381.1 (140.4)
Time domain OCT				
*N*	96	92		—
Mean (SD)	341.6 (129.0)	342.0 (124.7)		—
Spectral domain OCT				
*N*	812	805		—
Mean (SD)	389.3 (145.2)	381.5 (137.6)		—
*n* (%)				
≤300 *μ*m	298 (32.7)	308 (34.2)		606 (33.4)
>300 *μ*m	614 (67.3)	593 (65.8)		1207 (66.6)
Total	912 (100.0)	901 (100.0)		1813 (100.0)

^a^Due to the lack of data in 1 patient, analyses were for 1888 eyes in 944 patients.

^b^Due to the noninterventional design, data were available only for patients who had the test.

**Table 4:** Planned treatments for the patients.

Treatment, *n* (%)	Right eye	Left eye	Total
Anti-VEGF + laser	264 (36.8)	174 (24.6)	438 (30.8)
Anti-VEGF	240 (33.5)	213 (30.1)	453 (31.8)
Laser	178 (24.8)	279 (39.5)	457 (32.1)
Steroid + laser	13 (1.8)	10 (1.4)	23 (1.6)
Steroid	4 (0.6)	6 (0.8)	9 (0.6)
Vitrectomy	3 (0.4)	3 (0.4)	7 (0.5)
Steroid + anti-VEGF	1 (0.1)	1 (0.1)	2 (0.1)
Other	14 (2.0)	21 (3.0)	35 (2.5)
Total	717 (100.0)	707 (100.0)	1424 (100.0)

**Table 5:** Clinical characteristics of patients who did or did not receive antidiabetic treatment.

	Antidiabetic treatment		*p* value
	No	Yes	
*Type of diabetes, n (%)*			
Type 1	1 (3.1)	31 (96.9)	**0.005**
Type 2	195 (24.9)	588 (74.1)	
*Time from onset of diabetes to DME, n (%)*			
Less than 5 years	27 (30.3)	62 (69.7)	**<0.001**
5–9 years	73 (40.8)	106 (59.2)	
10–14 years	70 (29.2)	170 (70.8)	
15–19 years	17 (9.9)	154 (90.1)	
20 years +	9 (6.6)	127 (93.4)	
*Smoking status, n (%)*			
Smoking	6 (9.1)	60 (90.9)	**<0.001**
Used to smoke	12 (6.2)	183 (93.8)	
Never smoked	178 (32.1)	376 (67.9)	
*Hypertension*			
Not present	151 (38.9)	237 (61.1)	**<0.001**
Present	45 (10.5)	382 (89.5)	
*Dyslipidemia*			
Not present	171 (32.3)	358 (67.7)	**<0.001**
Present	25 (8.7)	261 (91.3)	
*DME type: right eye, n (%)*			
Diffuse	121 (34.5)	230 (65.5)	**<0.001**
Focal	56 (19.3)	234 (80.7)	
Mixed	10 (12.5)	70 (87.5)	
*DME type: left eye, n (%)*			
Diffuse	70 (24.8)	212 (75.2)	0.269
Focal	99 (29.1)	241 (70.9)	
Mixed	16 (21.3)	59 (78.7)	
*CMT: right eye, n (%)*			
≤300 *μ*m	47 (17.4)	222 (82.6)	**0.001**
>300 *μ*m	147 (28.4)	371 (71.6)	
*CMT: left eye, n (%)*			
≤300 *μ*m	42 (15.1)	237 (84.9)	**<0.001**
>300 *μ*m	149 (29.9)	349 (70.1)	
*CMT: right eye; median (min–max)*	376.5 (160–840)	342 (146–999)	**0.002**
*CMT: left eye; median (min–max)*	370 (201–780)	329 (148–999)	**<0.001**

CMT: central macular thickness; DME: diabetic macular edema.

Mann-Whitney *U*, chi-square test, and Fisher's exact test were used with Bonferroni correction.
